# An fMRI Study of Response and Semantic Conflict in the Stroop Task

**DOI:** 10.3389/fpsyg.2019.02426

**Published:** 2019-10-31

**Authors:** Benjamin A. Parris, Michael G. Wadsley, Nabil Hasshim, Abdelmalek Benattayallah, Maria Augustinova, Ludovic Ferrand

**Affiliations:** ^1^Department of Psychology, Bournemouth University, Poole, United Kingdom; ^2^School of Psychology, University College Dublin, Dublin, Ireland; ^3^Exeter MR Research Centre, University of Exeter, Exeter, United Kingdom; ^4^Normandie Université, UNIROUEN, CRFDP, Rouen, France; ^5^Université Clermont Auvergne, CNRS LAPSCO, Clermont-Ferrand, France

**Keywords:** task conflict, semantic conflict, response conflict, fMRI, selective attention, Stroop 2-1 mapping, Stroop

## Abstract

An enduring question in selective attention research is whether we can successfully ignore an irrelevant stimulus and at what point in the stream of processing we are able to select the appropriate source of information. Using methods informed by recent research on the varieties of conflict in the Stroop task the present study provides evidence for specialized functions of regions of the frontoparietal network in processing response and semantic conflict during Stroop task performance. Specifically, we used trial types and orthogonal contrasts thought to better independently measure response and semantic conflict and we presented the trial types in pure blocks to maximize response conflict and therefore better distinguish between the conflict types. Our data indicate that the left inferior PFC plays an important role in the processing of both response and semantic (or stimulus) conflict, whilst regions of the left parietal cortex (BA40) play an accompanying role in response, but not semantic, conflict processing. Moreover, our study reports a role for the right mediodorsal thalamus in processing semantic, but not response, conflict. In none of our comparisons did we observe activity in the anterior cingulate cortex (ACC), a finding we ascribe to the use of blocked trial type presentation and one that has implications for theories of ACC function.

## Introduction

The Stroop task (Stroop, 1935; MacLeod, 1991) has been referred to as the “gold standard” measure of selective attention (MacLeod, 1992). It elicits cognitive conflict by presenting two sources of information one of which is the relevant to-be-identified color and the other an irrelevant word and must be ignored. The Stroop interference effect refers to the finding that naming aloud the color that a word is printed in takes longer when the word denotes a different color (e.g., the word *red* displayed in blue font; an incongruent trial) compared to a baseline control condition (e.g., *top* in red or *xxxx* in red). The Stroop facilitation effect refers to the finding that naming aloud the color that a word is printed in is faster when the word denotes the same color (e.g., the word *red* displayed in red font; an congruent trial) compared to a baseline control condition. Influential models of Stroop task performance attribute Stroop effects to response level competition (or convergence in the case of facilitation; Cohen et al., 1990; Roelofs, 2003). Yet, more recent lines of research argue that these effects result from several distinct types of competition. Therefore, the present paper addressed just this issue by investigating the neural substrates of multiple sources of competition in the Stroop task.

### The Neural Substrates of Stroop Task Performance

The common implementation of the Stroop task involves incongruent, congruent and color neutral trials and imaging studies employing some or all of these conditions have consistently and mainly implicated left lateral prefrontal (particularly inferior frontal regions of BA44/45/47) and left parietal cortices in Stroop task performance (e.g., Bench et al., 1993; Khorram-Sefat et al., 1996; Peterson et al., 1999; Zysset et al., 2001; Adleman et al., 2002; Mead et al., 2002; Langenecker et al., 2004; Liu et al., 2004; Coderre et al., 2008; Song and Hakoda, 2015; Cipolotti et al., 2016). Many studies have also implicated the anterior cingulate cortex (ACC) in Stroop task performance (e.g., Bench et al., 1993; Peterson et al., 1999; Adleman et al., 2002; Langenecker et al., 2004; Liu et al., 2004; Coderre et al., 2008), although this is a matter of debate (e.g., Khorram-Sefat et al., 1996; Zysset et al., 2001; Mead et al., 2002; Roelofs et al., 2006; Aarts et al., 2008; Song and Hakoda, 2015).

An influential model (Botvinick et al., 2001) posits that the ACC is responsible for detecting the presence of response conflict between competing representations and consequently engages the DLPFC to impose cognitive control by biasing information in posterior cortices to resolve conflict (see also Miller and Cohen, 2001; van Veen and Carter, 2002). The parietal regions in contrast are thought to represent stimulus-response mappings or to be involved in visuospatial selection, and thus play a role in conflict resolution Casey et al., 2000; Rushworth et al., 2001; Bunge et al., 2002).

The Cascade-of-Control model (Banich, 2009, 2019) is another model of the neural substrates of Stroop task performance based on a series of studies investigating control in Stroop-like tasks (e.g., Banich et al. (2000a, b); Milham et al., 2002; Compton et al., 2003; Liu et al., 2006; Mackiewicz Seghete et al., 2017). According to this model, posterior portions of the lateral prefrontal cortex, particularly portions of the inferior frontal gyrus, are responsible for setting the attentional set in the Stroop task, meaning that it can upregulate color processing and/or downregulate word processing, prior even to stimulus onset (proactive control). The posterior PFC will send signals to posterior brain regions to ensure the biasing of relevant information over irrelevant information. Mid dorsolateral prefrontal cortex (DPLFC) is purported to be responsible for selecting relevant information in working memory on the presentation of the Stroop stimulus. If the prefrontal regions do not do as good a job as they could posterior and dorsal ACC regions are argued to play a role in late stage, response-related selection. Finally, consistent with the conflicting monitoring model of Botvinick et al. (2001), more rostral regions of the ACC are responsible for response evaluation and sending signals back to the DLPFC so that it can adjust the strength of its involvement. An important concept with the Cascade-of-Control model is that the involvement of certain regions, particularly the ACC, depends on how well the early selection regions do their jobs. Moreover, according to the model the posterior and dorsal ACC are thought to play a role only in response conflict resolution and not conflict of other types such as the conflict between semantic representations activated by the dimensions of the Stroop stimulus (semantic conflict) or the conflict between the exogenously activated task set for reading and the endogenously activated task set for color classification (task conflict).

### Dissociating Response and Semantic Conflict

It is notable that few studies have attempted to decompose Stroop effects into their components. Stroop interference for example has been shown to comprise conflict at a variety of different levels of processing (Augustinova et al., 2019; Ferrand et al., 2019; for a review see Parris, Hasshim, Wadsley, Augustinova, and Ferrand, under review). Doing so not only refines our understanding of the mechanisms of selective attention but also has the potential to elucidate the functions of associated brain regions. Indeed, it has been postulated that different regions of the ACC detect differential types of conflict (e.g., response and semantic conflict; van Veen and Carter, 2005) which then engage separate regions of the PFC to independently resolve semantic (superior PFC) and response conflict (inferior PFC). In contrast, the results from another study suggest that PFC activity dissociates by hemisphere (Milham et al., 2001). Milham et al. report that right PFC is responsible for resolving response conflict while left PFC is responsible for resolving semantic conflict. van Veen and Carter (2005) also reported parietal activation to semantic conflict only, consistent with the notion that it plays a role in maintaining task-relevant response mappings. Milham et al. in contrast reported parietal activity to both response (superior parietal lobe) and semantic (inferior parietal lobe) conflict.

van Veen and Carter (2005) noted that the differences between their study and that of Milham et al. might be due to the way response and semantic conflict were measured (see below for more detail). Recent research concurs with this conclusion. The aim of the present study was to investigate the neural regions involved in processing different types of conflict using methods informed by recent research (Augustinova and Ferrand, 2014; Hasshim and Parris, 2014, 2015, 2018; Levin and Tzelgov, 2016). Below we describe and critically evaluate the methods employed thus far in the study of the neural correlates of response and semantic conflict.

### The 2:1 Color-Response Mapping Paradigm

In their study van Veen and Carter (2005) employed the 2:1 color-response mapping paradigm. First introduced by De Houwer (2003) this method maps two color responses to the same response button, which allows for a distinction between stimulus-stimulus (semantic) and stimulus-response (response) conflict. By mapping two response options onto the same response key (e.g., both “blue” and “yellow” are assigned to the “z” key) any interference during same-response trials (e.g., when “blue” is printed in yellow) is thought to involve only semantic conflict. Any additional interference on incongruent trials (e.g., when “red” is printed in yellow and where both “red” and “yellow” are assigned to different response keys) is taken as an index of response conflict. Performance on congruent trials is compared to performance on same-response incongruent trials to reveal interference that can be attributed to semantic conflict, whereas a different-response incongruent – same-response incongruent trial comparison is taken as in index of interference due to response conflict. Thus, the main advantage of using same-response incongruent trials as an index of semantic conflict is that it claims to be able to remove all the influence of response competition (De Houwer, 2003; Schmidt and Cheesman, 2005).

Using a Flanker task, van Veen et al. (2001) tested 12 participants using same-response and different-response incongruent trials to investigate the response of the ACC to response and stimulus conflict. They reported that the ACC was active only when response conflict was present, and that stimulus conflict activated the left inferior frontal gyrus. In their follow up study using the Stroop task with 14 participants, van Veen and Carter (2005) observed no overlap of activation between semantic and response conflict. They showed that semantic conflict activated dorso-lateral prefrontal cortex (DLPFC: BA8/9), posterior parietal cortex (PPC: BA40) and the (ACC: BA32/6), whereas response conflict activated more inferior lateral prefrontal cortex (BA9/44/45/46), left premotor areas (BA6) and regions of the ACC (BA24/32) more anterior and ventral to that activated by semantic conflict (see also Chen et al., 2013, and Kim et al., 2010, for replications of this finding). This finding of ACC activation to semantic conflict conflicts with the Cascade-of-Control model (Banich, 2009, 2019). The authors argued that their findings were consistent with and extended the conflict monitoring account (Botvinick et al., 2001) by showing the involvement of separable regions of the ACC in monitoring for different types of conflict. Thus, using the 2:1 color-response mapping method, response and semantic conflict have been dissociated at the neural level. However, despite providing a seemingly convenient way of separating these different forms of conflict, Hasshim and Parris (2014, 2015) have shown, using both RT and pupillometry as dependent variables, that same-response trials do not differ from non-color word neutral trials (e.g., *top* in red) questioning their utility in dissociating response and semantic conflict (see Parris et al., under review, for a review and fuller discussion of this issue).

### Non-response Set Trials

The only other trial type that has been used to dissociate the neural substrates of response and semantic conflict is non-response set trials (Milham et al., 2001). Non-response set trials are trials on which the irrelevant color word used is not one of the possible response colors (e.g., the word “orange” in blue, where orange is not a possible response option and blue is; originally introduced by Klein, 1964). Since the non-response set color word will activate color-processing systems, interference on such trials can be taken as evidence for conflict occurring at the semantic level. These trials should in theory remove the influence of response conflict, as the irrelevant color-word is not a possible response option, and thus conflict at the response level is not present. The difference in performance between the non-response set trials and a neutral word baseline condition (e.g., the word “table” in red) is taken as evidence of interference caused by the semantic processing of the irrelevant color word. Whereas response conflict can be isolated by comparing the difference between the performance on incongruent trials and the non-response set trials. This index of response conflict is referred to as the *response set effect* and describes the interference that is a result of the irrelevant word denoting a color that is also a possible response option.

Milham et al. (2001) investigated the neural substrates of response and non-response-related conflict using response- and non-response set trials, but blocked stimulus presentation such that a block contained either response set trials and neutral trials or non-response set trials and neutral trials (see also Milham et al., 2003). Consistent with van Veen et al. (2001), but inconsistent with van Veen and Carter (2005) they reported ACC activation to response conflict but no ACC activation to non-response conflict. They also reported that both left and right PFC were activated by response conflict, but only left PFC was activated by semantic conflict, a finding that is inconsistent with previous imaging studies. The lack of ACC activation to semantic conflict indicates that the theorized conflict monitoring processes (Botvinick et al., 2001) are not processing all types of conflict, which is consistent with the Cascade-of-Control model (Banich, 2009, 2019).

Whilst the response set effect might provide a useful measure of response conflict, the magnitude of the response set effect has varied between studies. Noting this, Hasshim and Parris (2018) reported within-subjects experiments in which the trial types (e.g., response set, non-response set, neutral) were presented either in separate blocks (pure) or in blocks containing all trial types in a random order (mixed). They observed a decrease in RTs to response set trials when trials were presented in mixed blocks when compared to the RTs to response set trials in pure blocks. The findings demonstrate that presentation format modulates the magnitude of the response set effect, and thus response conflict, substantially reducing it when trials are presented in mixed blocks. In contrast, semantic conflict was not significantly affected by the manipulation. It is important for studies to consider how these manipulations may be used to maximize the detection of a response set effect (response conflict); all previous fMRI investigations of response and semantic conflict have employed mixed blocks. Hasshim and Parris (2018) results suggests that the use of pure blocks will enable a better index of response conflict. For this reason, in the present study we presented trial types in pure blocks. A further benefit of this approach is that blocked designs remain the most statistically powerful designs for fMRI experiments with the recommendation that each block should be between 16–40 s in duration (Bandettini and Cox, 2000). Moreover, the use of pure blocks also has potential implications for the role of the ACC in Stroop task performance and conflict processing.

### The Role of the ACC in Stroop Task Performance

As noted above, ACC activation has been observed in neuroimaging studies of the Stroop task (Bench et al., 1993; Peterson et al., 1999; Adleman et al., 2002; Langenecker et al., 2004; Liu et al., 2004; Coderre et al., 2008) and, as noted, has been theorized to have an important role in Stroop task performance, particularly in detecting response conflict (Botvinick et al., 2001; Banich, 2009, 2019) and have separable regions for detecting response and semantic conflict (van Veen and Carter, 2005; cf. Milham et al., 2001). However, the role of the ACC in the Stroop task has been debated (Botvinick et al., 2001; Fellows and Farah, 2005; Roelofs et al., 2006; Aarts et al., 2008) with some work showing that atrophy of the ACC has no effect on Stroop task performance (Swick and Jovanovic, 2002; Fellows and Farah, 2005). Importantly for present purposes, in a recent study Floden et al. (2011) showed that ACC involvement in Stroop task performance is substantially larger when trial types are presented randomly intermixed compared to when presented in pure blocks, which the authors tentatively argued supported the notion that ACC activation reflects arousal and not conflict monitoring. If trial type mixing were responsible for ACC activations observed in the Stroop task, we should see little to no ACC activation to response nor semantic conflict, which would contrast with findings showing separate regions of the ACC being involved in response and semantic conflict and with theories positing a role for the ACC in detecting conflict, especially since response conflict is maximized using pure block designs.

### Semantic-Associative Trials and the Orthogonality of Comparisons

A final method of dissociating response and semantic conflict is through the use of semantic-associative trials. In these trials the irrelevant words used are associatively related to the response colors (e.g., sky – blue, grass – green). This method of isolating semantic conflict was also first introduced by Klein (1964) and has since been used in many studies investigating semantic Stroop interference (Stirling, 1979; Sharma and McKenna, 1998; Risko et al., 2006; Augustinova and Ferrand, 2014; see also Neely and Kahan, 2001). This is important because having another well-validated way of separating response and semantic conflict permits us to address another issue with previous studies attempting to dissociate response and semantic conflict; and that is the issue of orthogonality of comparisons (Levin and Tzelgov, 2016). In all previous studies, the estimation of response conflict has been computed by comparing standard incongruent trials with the trial type used to index semantic conflict (e.g., same-response trials, non-response set trials). The trial type used to index semantic conflict has then been used again to compute semantic conflict against a neutral trial. This multiple use of a single trial type to compute the two different forms of conflict results in contaminated non-orthogonal measures (Levin and Tzelgov, 2016). To avoid this issue in the present study we compare standard incongruent trials with semantic-associative trials to get an index of response conflict, and non-response set and neutral trials to get a measure of semantic conflict.

### Task Conflict

Another form of conflict thought to contribute to Stroop effects is task conflict. The presence of task conflict was first proposed in MacLeod and MacDonald’s (2000) review of brain imaging studies. The authors proposed its existence because the ACC appeared to be more activated by incongruent and congruent stimuli when compared to repeated letter neutral stimuli (e.g., xxxx). They suggested that increased ACC activation by congruent and incongruent stimuli is likely an expression of the task conflict caused by the automatically activated, irrelevant reading task and the intentionally activated color identification task. This suggestion was recently supported in a computational model of task conflict (Kalanthroff et al., 2018) and in an fMRI study of a task switching task that also reported a dissociation between response and task conflict in the ACC (Desmet et al., 2011). However, no study has yet sought to confirm this hypothesis in a neuroimaging study of the Stroop task itself.

Since task conflict is produced by the activation of the mental machinery used to read, interference at this level occurs with any stimulus that is found in the mental lexicon. In line with this any readable letter string should produce more interference than any unreadable, non-word letter string. Previous studies have used this logic in order to isolate task conflict from informational conflict (e.g., Entel and Tzelgov, 2018). Since both congruent and incongruent trials produce task conflict, trials consisting of repeated letters or symbols (e.g., xxxx or ####) have been introduced as a baseline (e.g., Monsell et al., 2001; Kalanthroff et al., 2015; Entel and Tzelgov, 2018). However, non-word letter strings (e.g., xxxx) are still likely to activate letter reading processes which may produce conflict between word processing and color processing to some extent. Levin and Tzelgov (2016) used unreadable common shapes instead of letter strings to measure task conflict since using repeated letters might activate the task set for word reading to some extent. This is a potentially important modification, but one issue with the use of common shapes is that the use of common, unreadable but nameable shapes might well have activated a shape naming task set that could interfere with the color naming task set. Therefore, in contrast to Levin and Tzelgov, in the present study we employed uncommon, unnameable shapes to prevent a shape-naming task set from interfering in the color naming process. However, to foreshadow our results an initial manipulation check revealed that our unnameable shape baseline was indistinguishable from our neutral baseline in both the RT and neutral data. Furthermore, in a separate unpublished oculomotor Stroop study run alongside the present study, these stimuli produced longer RTs than even our standard incongruent condition. It is unclear why this condition presented such a challenge for our participants, but beyond reporting this simple analysis we draw no conclusions regarding task conflict.

### Summary

Using the 2:1 color response mapping paradigm, both van Veen and Carter (2005) and Chen et al. (2013) showed that semantic conflict activated DLPFC, PPC and the ACC, whereas response conflict activated more inferior lateral PFC, left premotor areas and regions of the ACC that were more anterior and ventral to that activated by semantic conflict. These findings are consistent not only with a monitoring role for the ACC and a conflict resolution role for lateral PFC regions, they also suggest that distinct areas of both regions separately process response and semantic conflict. However, the employment of the 2:1 paradigm renders the interpretation of their data less clear. Using non-response set trials, Milham et al. (2001) reported ACC and specifically right PFC activation to response conflict, but activity in left PFC to both response and semantic conflict. This finding is consistent with a role for the ACC in monitoring for response conflict, but not semantic conflict. However, both studies mixed trial types which could be responsible for ACC activation during Stroop task performance (Floden et al., 2011) and furthermore does not maximize response conflict (Hasshim and Parris, 2018). Moreover, they employed non-orthogonal contrasts in their measures of semantic and response conflict. Finally, task conflict has been hypothesized to be reflected in ACC activity but no study has yet provided supporting evidence for this.

**FIGURE 1:**
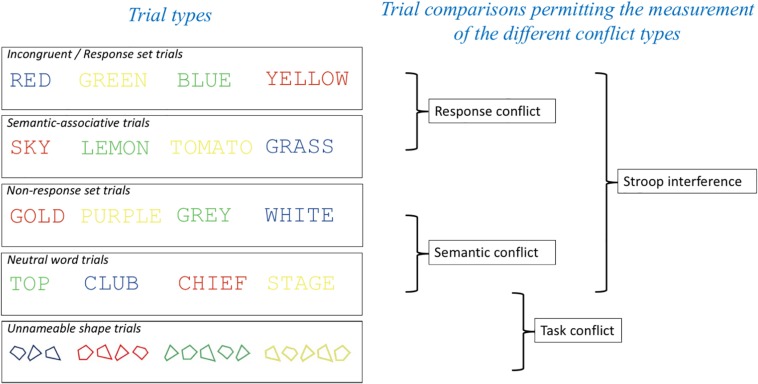
Trial types employed and comparisons made in the experiment to enable the indexing of the different conflict types in the Stroop task. Having two separate trial types that permit the measurement of semantic conflict without response conflict (Semantic-associative and Non-response set trials) meant that our measurements of response conflict [Incongruent (response set) – Semantic-associative] and semantic conflict (Non-response set and Neutral word trials) were orthogonal to each other.

In the present study, we investigated the neural substrates of response, semantic and task conflict by presenting five different trial types in pure blocks. The following trial types were employed in this experiment: Response set (standard incongruent) trials, non-response set trials, semantic-associative trials, color neutral trials and non-nameable shapes. However, following recommendations from Levin and Tzelgov (2016) for ensuring orthogonality of comparisons in the Stroop task we made the following comparisons to index response and semantic conflict: (1) For semantic conflict we compared performance on non-response set trials and neutral trials; (2) Response conflict was isolated using an incongruent (response set) vs. semantic associative condition comparison. Finally, for comparison with the neuroimaging studies of the general Stroop effect (e.g., Bench et al., 1993; Khorram-Sefat et al., 1996; Peterson et al., 1999; Zysset et al., 2001; Adleman et al., 2002; Mead et al., 2002; Langenecker et al., 2004; Liu et al., 2004; Coderre et al., 2008; Song and Hakoda, 2015; Cipolotti et al., 2016) we also accepted non-orthogonality when comparing incongruent and neutral trials (see Figure 1).

## Methods

### Participants

Twenty participants (14 female, M_age_ = 23.90, SD = 7.40), recruited from Bournemouth University’s staff and student populations, were tested. All participants were 18–45 years old, fluent in English and had normal or corrected-to-normal vision, as well as normal color vision. Each participant received £10 and a copy of their structural brain scan for participating. The study was approved by the Bournemouth University research ethics committee, and all subjects provided fully informed consent to participate.

### Materials and Measures

#### Stimuli

Twelve unique stimuli were used for each of our five conditions (unnameable shape trials, neutral word trials, semantic-associative trials, non-response set trials, and incongruent trials). Items were presented individually in uppercase Courier New font, size 42, in the center of the screen on a black background. Four irregular shapes were used to make up four unique shape string trials (matched to the word length of the colors in the response set). The shapes consisted of two irregular quadrilaterals and two irregular pentagons. Other trials consisted of: neutral non-color words: TOP, CLUB, STAGE, CHIEF; color-associated words: SKY, TOMATO, LEMON, GRASS; color words (non-response): PURPLE, GOLD, WHITE, GRAY; incongruent color words: RED, BLUE, GREEN, YELLOW. Color-associated words were always presented in an incongruent color (e.g., “grass” would be presented in red, blue or yellow as opposed to green). Participants responded to the colors red (RGB: 255; 0; 0), blue (RGB: 0; 32; 96), green (RGB: 0; 176; 80), and yellow (RGB: 255; 255; 0) by pressing the corresponding key on a Cedrus response box.

### Procedure

After informed consent had been obtained participants entered the MRI scanner and completed practice trials while a structural scan was performed. The practice trials consisted of 32 color patches (8 of each response color: red, blue, green and yellow) presented in a random order. Participants responded to the color using a Cedrus response box. After the practice trials participants completed the 600 experimental trials whilst BOLD activation was recorded. Participants were instructed to respond as quickly and as accurately as possible to the color of each stimulus whilst ignoring the meaning of the irrelevant word.

OpenSesame 3.2 software (Mathôt et al., 2012) was used to administer the Stroop task. The stimuli were presented in pure blocks containing all 12 stimuli for each condition. Each run contained the five conditions, with each condition presented in a random order for each new run. Each run was repeated 10 times, meaning that each participant completed a total of 600 trials (120 trials per condition), giving us more than the recommend 1600 observations per condition across all subjects (Brysbaert and Stevens, 2018). Each trial began with a fixation cross for 500 ms. The stimuli were then presented for 1000 ms followed by an inter-stimulus interval of 1000 ms during which a black screen was shown. After each block of 12 stimuli a break occurred for 10 s. Each testing session lasted approximately 45 min.

### Image Acquisition

Scanning was performed on a 1.5T Philips Intera magnet with standard RF head coil at the *Exeter MR Research Centre, University of Exeter, United Kingdom*. A T_2_^∗^-weighted echo planar imaging (EPI) sequence was used (TR = 2300 ms, TE = 45 ms, flip angle = 90°, 30 oblique transverse slices in ascending order and matrix size = 3 × 3 × 3.5 mm). A total of 880 volumes were acquired for each subject. Participants were able to view the stimuli on a screen placed at the foot of the scanner via a mirror mounted on the head coil. Between each block there was a break for 10 s to allow the BOLD signal to return to baseline.

### Image Analysis

Data were analyzed using SPM12 Software^1^. The fMRI images were pre-processed -realigned, sliced timed (ascending sequence, 30 slices, TR = 2300 ms), normalized and smoothed (to 8 mm). Statistical regressors were generated by convolving a canonical hemodynamic response function with a series of discrete event onset times for blocks (30 s duration) corresponding to the presentation of stimuli in the unnameable shapes, neutral word, semantic-associative, non-response set and incongruent conditions. A general linear model approach was used to estimate parameter values for each regressor. Having created a series of t-contrast images for each effect for each subject, the contrast images were entered into a 2nd level (“random effects”) analysis consisting of one-sample *t*-tests with a hypothesized mean of 0 (thresholded at *p* = 0.001). Following Parris et al. (under review), and to further protect against the probability of type 1 error, we employed an extent voxel threshold cut-off of 30. This combination of intensity and extent thresholds produces a per voxel false positive probability of < 0.000001 (Forman et al., 1995). Two sample repeated measures *t*-tests with a statistical threshold of *p* < 0.001, uncorrected, and a voxel cluster size threshold of 30 were also performed for each of the planned comparisons. In order to determine the site of activation, MNI (SPM) coordinates were converted to Talairach coordinates using BioimageSuite^2^ (Lacadie et al., 2008).

## Results

### Analysis of Mean Response Times

The mean RTs of correct responses for each participant in each condition were subjected to a one-way repeated measures ANOVA. All RT outliers (RTs < 300 ms) were excluded from the analysis. In total seven trials were excluded as outliers (2 unnameable shapes, 1 semantic associate, 1 non-response, and 3 incongruent trials). The mean RTs of each experimental condition are summarized in Table 1.

**TABLE 1:** Mean response latencies (ms) per condition.

	**Shapes**	**NW**	**SA**	**NRS**	**Incongruent**
RTs (ms)	638.86 (55.56)	634.11 (65.31)	641.95 (68.68)	650.82 (75.75)	655.91 (77.63)

*“NW” refers to neutral words. “SA” refers to semantic associates. “NRS” refers to non-response set. SD is presented between parentheses.*

Mauchly’s test indicated that the assumption of sphericity had been violated χ^2^(9) = 25.13, *p* = 0.003, therefore the degrees of freedom were corrected using Greenhouse-Geisser estimates of sphericity (ε = 0.56). The results of the one-way repeated measures ANOVA revealed that the main effect of condition was significant *F*(2.23, 42.40) = 4.59, *p* = 0.013, ${\text{η}}_{\text{p}}^{2}$ = 0.195. Therefore, follow up pairwise comparisons were conducted for each of our planned comparisons. The comparison for task conflict (neutral words vs. unnameable shapes) revealed a non-significant difference between conditions [*t*(19) = −0.98, *p* = 0.340]. The comparison for semantic conflict revealed a significant semantic Stroop effect [*t*(19) = 3.04, *p* = 0.007]. The comparison for response conflict was also significant [*t*(19) = 2.38, *p* = 0.028]. Finally, an overall Stroop effect was observed using an incongruent vs. neutral word comparison [*t*(19) = 3.14, *p* = 0.005].

### Analysis of Errors

Errors, including incorrect responses and time-out errors, accounted on average for 12.63% of the trials (unnameable shapes 12.71%: neutral words 11.08%; semantic associates 12.17%; non-response set 12.33%; incongruent 14.71%), which is similar to error rates seen in other fMRI assays (e.g., van Veen and Carter, 2005). An omnibus ANOVA for error rates across the five conditions was conducted. Mauchly’s test indicated that the assumption of sphericity had been violated χ^2^(9) = 36.99, *p* = 0.001, therefore the degrees of freedom were corrected using Greenhouse-Geisser estimates of sphericity (ε = 0.49). The results showed that the effect of condition on the rate of response errors was non-significant *F*(1.98, 37.54) = 2.39, *p* = 0.106, ${\text{η}}_{\text{p}}^{2}$ = 0.112. Because our ANOVA revealed no significant effect of condition on error rates, follow-up pairwise comparisons between conditions were not carried out.

### fMRI Data

**FIGURE 2:**
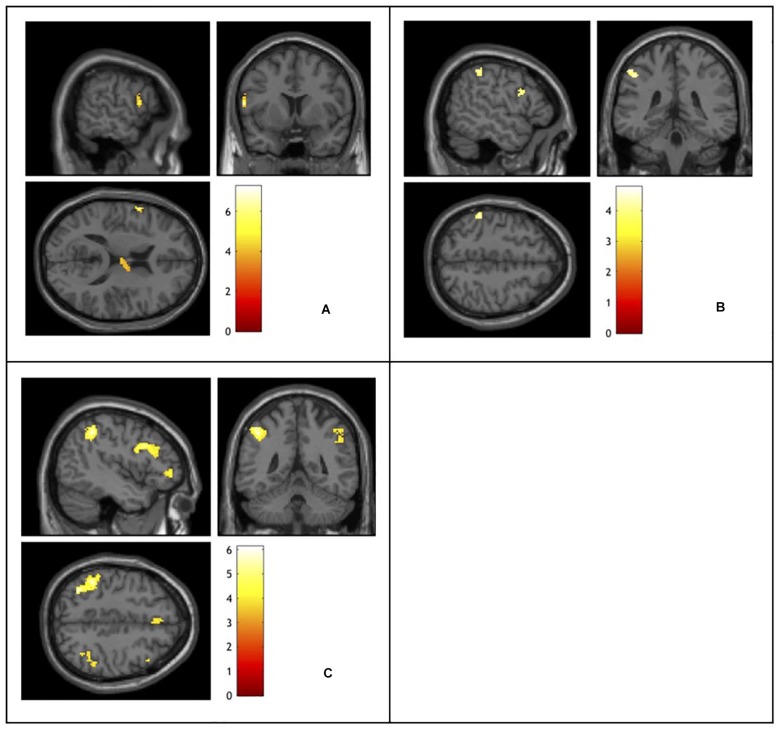
Functional magnetic resonance imaging activation elicited by: **(A)** semantic conflict indexed using a non-response set – neutral words contrast. **(B)** Response conflict indexed using an incongruent – semantic associates contrast. **(C)** Stroop interference indexed using an incongruent – neutral words contrast. Activation color represents *t*-values.

Analysis of the fMRI data revealed different patterns of brain activity in response to the different types of conflict indexed (see Table 2 and Figure 2). Planned contrasts were carried out to reveal the brain regions that elicited activity in response to each of the types of conflict. The contrast for task conflict did not show any significant sites of activation. Compared to neutral word trials, non-response set trials elicited a significant cluster of activation in the left inferior frontal gyrus (BA44). Semantic conflict also led to a significant cluster of activation in the right thalamus. The comparison between incongruent and semantic associate trials, our index for response conflict, revealed activity in the left parietal (BA40) and prefrontal cortices (BA44/9). Finally, the incongruent – neutral word contrast revealed the brain regions recruited by the overall Stroop interference effect. The largest clusters of activation were found bilaterally in the dorso-lateral PFC (BA44/8/9/10) and the left parietal cortex (BA40), as well as activation within the right mediodorsal nucleus of the thalamus. Importantly, no activation was observed within the ACC in any of the contrasts even when the alpha and cluster thresholds were lowered to match that of previous studies that do report ACC activation (Milham et al., 2001; van Veen and Carter, 2005), and this is despite the present study involving more participants, with more trials per condition, and using the more powerful block design.

**TABLE 2:** Activated areas in response to each of the components of Stroop interference.

		**Talairach coordinates**				
**Cluster region**	**BA**	***X***	***Y***	***Z***	**Size**	**Z score**
**Task Conflict (NW – US)**						
No significant activation	n/a	n/a	n/a	n/a	n/a	n/a
**Semantic conflict (NRS– NW)**						
L Inferior frontal gyrus	44	−57	5	13	40	4.95
R thalamus proper	50	4	−12	15	96	3.57
**Response conflict (I – SA)**						
L inferior parietal lobule	40	−48	−35	43	72	3.81
L inferior frontal gyrus	44	−54	13	27	46	3.61
L middle frontal gyrus	9	−33	35	21	46	3.47
**Overall conflict (I – NW)**						
L inferior parietal lobule	40	−44	−43	43	530	4.49
L inferior frontal gyrus	44	−52	23	27	544	4.36
L superior frontal gyrus	8	−6	34	33	151	4.06
L middle frontal gyrus	10	−37	40	0	86	3.92
R inferior frontal gyrus	44	52	12	27	50	3.74
R inferior parietal lobule	40	50	−39	36	155	3.73
R superior parietal lobule		31	−55	37	71	3.69
R middle frontal gyrus	9	45	32	21	128	3.61
R middle frontal gyrus	10	33	45	5	56	3.55
R superior frontal gyrus	8	45	22	44	51	3.45
R thalamus proper	50	11	−9	15	48	3.42

*“US” refers to unnameable shapes. “NW” refers to neutral words. “SA” refers to semantic associates. “NRS” refers to non-response set. “I” refers to incongruent. The normalized voxel size was 2 × 2 × 2 mm. Only clusters of 30 voxels or greater are presented.*

## Discussion

The aim of the present study was to investigate the neural substrates of response, semantic and task conflict using methods informed by recent research (Augustinova and Ferrand, 2014; Hasshim and Parris, 2014, 2015, 2018; Levin and Tzelgov, 2016). Following critical evaluation of previous methods employed in influential neuroimaging investigations (e.g., Milham et al., 2001; van Veen and Carter, 2005) we used trial types thought to better independently measure response and semantic conflict (see Parris et al., under review, for a review) and unlike previous studies computed orthogonal contrasts. Furthermore, we presented the trial types in pure blocks to both maximize response conflict and assess the role of the ACC in Stroop task performance. Finally, our study also included a measure of task conflict. In what follows we summarize our findings by considering their implications for each of the regions associated with Stroop task performance.

### Anterior Cingulate Cortex

An important finding to note first, since it applies to all comparisons made, is that we observed no ACC activations in any of our contrasts. This is a notable difference in reported findings between the present study and all previous studies of the neural substrates of Stroop task performance. This held even when reducing the threshold to that used in the other studies and despite testing more participants, having more trials per condition and using the more powerful block design. Indeed, we attribute this difference to the use of the block design (Floden et al., 2011). Floden et al. (2011) compared blocked and mixed designs and observed substantially reduced ACC activation in the blocked trials. This led the authors to conclude that ACC activation represents arousal and not conflict monitoring. Whilst our data do not allow us to conclude in favor of an arousal function of the ACC, this finding strongly contrasts with a the role of the ACC in conflict monitoring (e.g., Botvinick et al., 2001; Fellows and Farah, 2005; Roelofs et al., 2006; Aarts et al., 2008), and with the notion that separate regions of the ACC detect different forms of conflict (van Veen and Carter, 2005). However, this finding does not necessarily contradict the Cascade-of-Control model (Banich, 2009, 2019). The Cascade-of-Control model predicts a role for posterior and dorsal ACC in late stage, response selection, and more rostral ACC in conflict monitoring. Uniquely, however, it stipulates that the role these ACC regions play depends on how well the earlier selection regions of the PFC perform their role. Conceivably, presenting the trials in pure blocks, enables better proactive control by the inferior frontal gyrus, a key region of activation in the present study, mitigating the role of ACC regions. Nevertheless, the Cascade-of-Control model would predict posterior and ACC activation specifically for response conflict, which we isolated and for which we do not observe ACC activation.

As foreshadowed in the introduction our unnameable shape condition produced RTs equivalent to those in the neutral condition, which means we are unable to clarify the role of the ACC in this form of conflict. In order to index task conflict (the conflict that arises from reading the irrelevant word dimension of a Stroop stimulus) we proposed that unnameable irregular shapes would provide us with the most suitable baseline condition to compare against readable neutral word trials. Unexpectedly our data showed that the shape trials produced longer RTs and more response errors than neutral word trials and thus we were unable to demonstrate evidence for the effect of task conflict using this comparison. And whilst it has been convincingly argued that corroborative RT data is not necessarily needed to interpret fMRI data (Wilkinson and Halligan, 2004), it was also the case that the shape vs. neutral trial comparison in the fMRI data produced no significant activation sites in a whole brain analyses. Neither our data, nor existing literature, permits us to interpret the finding. We have subsequently observed similar RT findings in some unpublished data from an oculomotor study suggesting that unnameable shape trials are hard for participants to color name. More research is needed to understand this effect, but for now the results from the current study do not permit us to conclude anything regarding the neural substrates of task conflict.

### Prefrontal Cortex

The neural activations reported for the standard incongruent (response set) trials vs. neutral trial comparison largely reflects a combination of the activations for response and semantic conflict. Whilst this comparison revealed more bilateral activations compared to the generally more left-sided activations seen in the response and semantic conflict analyses, the larger activation clusters are in the left hemisphere. The largest clusters of activations for the overall Stroop effect were in the left inferior frontal gyrus (BA44) consistent with a role for this region in setting the attentional set and biasing activation toward the color dimension and away from the word dimension of the Stroop stimulus (Botvinick et al., 2001; Banich, 2009, 2019). Mid and superior dorsal PFC regions were also more greatly activated by incongruent than neutral trials consistent with a role for these regions in selected the relevant dimension of the Stroop stimulus (Banich, 2009, 2019). Our data do not, however, permit us to conclude in favor of the dissociated roles of the inferior and mid PFC regions posited by the Cascade-of-Control model.

In terms of neural activations to response conflict we observed activity in the left middle and inferior frontal gyri (BA9/44). The finding of an association between the left IFG and response conflict is consistent with a previous finding (van Veen and Carter, 2005), although it has more frequently been associated with semantic conflict (Milham et al., 2001; van Veen et al., 2001; Chen et al., 2013), but is inconsistent with the Cascade-of-Control model (Banich, 2009, 2019), which predicts this region is an area of early selection, not late, response selection, which the model places in the ACC. An association between the left middle frontal gyrus (BA9) and response conflict is more consistent with previous research (Milham et al., 2001; van Veen et al., 2001; Chen et al., 2013), but is somewhat inconsistent with the Cascade-of-Control model since according the model the PFC is responsible for early selection, although it is unclear whether the model removes a role completely for mid PFC regions in response conflict processing. However, in two of those studies (van Veen et al., 2001; Chen et al., 2013), same-response trials were used to dissociate response and semantic conflict. Given the findings of Hasshim and Parris (2014, 2015) the findings from these two studies might be better interpreted as being the equivalent of an incongruent and neutral trial comparison and not therefore isolated response conflict. Having used a better measure of response conflict the present study presents more reliable findings as to the neural substrates of response conflict.

The non-response set trial vs. neutral trial comparison indexing semantic conflict revealed activations in the left inferior frontal gyrus (IFG; BA44). The finding of activation associated with the left IFG is consistent with all previous studies investigating the neural mechanisms of semantic conflict (Milham et al., 2001; van Veen and Carter, 2005; Chen et al., 2013), although in these previous studies this activation was unique to semantic conflict with the exception of van Veen and Carter (2005). Again though, as noted, two of the studies (van Veen and Carter, 2005; Chen et al., 2013) used same-response trials. Our data suggest that the IFG (BA44) plays an important role in processing both response and semantic conflict. Whilst we have argued that the former is inconsistent with the Cascade-of-Control model, a role for the IFG in semantic conflict processing is not. Semantic conflict occurs earlier than response conflict, and since the Cascade-of-Control model argues the IFG is involved in early selection once could consider this result consistent with the model. Notably, however, the model is unclear about the regions that are involved in the processing of semantic, and indeed all non-response, conflict.

### Parietal Lobe

The results of the incongruent vs., neutral comparison also concurs with many previous studies highlighting the importance of the parietal regions, in the left hemisphere in particular, in Stroop task performance (e.g., Bench et al., 1993; Khorram-Sefat et al., 1996; Peterson et al., 1999; Zysset et al., 2001; Adleman et al., 2002; Mead et al., 2002; Langenecker et al., 2004; Liu et al., 2004; Coderre et al., 2008; Song and Hakoda, 2015). These regions mainly comprise the frontoparietal network, the control network responsible for our ability to coordinate behavior in a goal-driven manner (Marek and Dosenbach, 2018), a region implicated in many tests of executive function.

One of the largest clusters of activations for the overall Stroop effect was in the left parietal lobe (BA40) which was also important in the processing of response, but not semantic, conflict in our data. Response conflict has been associated with the left parietal region (specifically BA40) in the present study and in Chen et al. (2013) and Milham et al. and in studies not employing the Stroop task (Wendelken et al., 2009), and is consistent with the notion that inferior parietal lobe (BA40) might be involved in the allocation of attention to different posterior processing streams to bias processing toward the relevant processing stream (e.g., color) to reduce conflict (Liu et al., 2004). Furthermore, the finding that it is not involved in semantic conflict is consistent with notion that the parietal role plays a role in representing stimulus-response mappings (Casey et al., 2000; Rushworth et al., 2001; Bunge et al., 2002).

Whilst neither the conflict monitoring nor Cascade-of-Control models focus on the role of the parietal lobe in accounting for Stroop task performance, Banich (2009; 2019) notes that the frontoparietal network is implicated in biasing processing in posterior color and word processing regions of the brain.

### Thalamus

Whilst not unprecedented (Peterson et al., 1999) activations of the thalamus are not often reported in fMRI studies of the Stroop task but this might be because of the Region of Interest approach taken by studies investigating response and semantic conflict whereby analysis is restricted to frontal and parietal regions (van Veen and Carter, 2005; Chen et al., 2013). However, the part of the thalamus activated by semantic conflict in the present study, the medio-dorsal nucleus, receives input from the lateral prefrontal cortex and forms part of the fronto-striatal system of reciprocal, cortical-subcortical loops (Alexander et al., 1986), and has been implicated in processing stimulus-response relationships (Parris et al., 2007) with a general role hypothesized to be in temporally extending the efficiency of the cortical networks involving the prefrontal cortex (Pergola et al., 2018). Moreover, smaller thalamic volume has been associated with slower RTs and poorer performance on the Stroop task (Van Der Elst et al., 2007; see also Hughes et al., 2012). Finally, and as already noted, no ACC activation was observed for semantic conflict, although this particular finding need not necessarily be attributed to the block design employed (Floden et al., 2011), given that lack of ACC activation to semantic conflict has been reported in two previous studies (Milham et al., 2001; van Veen et al., 2001).

## Conclusion

In conclusion, using methods informed by recent research on the varieties of conflict in the Stroop task (see Parris et al., under review, for a review) the present study provides evidence for specialized functions of regions of the frontoparietal network in Stroop task performance. Specifically, together with previous research our data indicate that the left inferior PFC plays an important role in the processing of both response and semantic conflict, a finding that is broadly consistent with other work (e.g., Milham et al., 2001) whilst regions of the left parietal cortex (BA40) play an accompanying role in response, but not semantic, conflict processing. Moreover, our study reports a role for the thalamus in processing semantic, but not response, conflict. Finally, in none of our comparisons did we observe activity in the ACC, a finding we ascribe to the use of blocked trial type presentation (Floden et al., 2011) and one that is inconsistent with the conflict monitoring model (Botvinick et al., 2001). Whilst our results do not fully support the Cascade-of-Control model (Banich, 2009, 2019), the model does potentially account for most of the findings presented herein.

## Data Availability Statement

The datasets generated for this study are available on request to the corresponding author.

## Ethics Statement

The studies involving human participants were reviewed and approved by the Bournemouth University Research Ethics Committee. The patients/participants provided their written informed consent to participate in this study.

## Author Contributions

BP was involved in the design and analysis of the study, and wrote the manuscript. MW was involved in the design, preparation, data collection, analysis, and wrote portions of the manuscript. NH was involved in the design and preparation of the study, and provided comments on earlier drafts of the manuscript. AB was involved in the preparation of the study, data collection, and analysis. MA was involved in the design of the study and provided comments on earlier drafts of the manuscript. LF was involved in the design of the study and provided comments on earlier drafts of the manuscript.

## Conflict of Interest

The authors declare that the research was conducted in the absence of any commercial or financial relationships that could be construed as a potential conflict of interest.

## Footnotes

1 www.fil.ion.ucl.ac.uk/spm

2 www.bioimagesuite.org
